# Intratubular penetration ability in the canal perimeter using HiFlow bioceramic sealer with warm obturation techniques and single cone

**DOI:** 10.4317/jced.59815

**Published:** 2022-08-01

**Authors:** Alberto Casino-Alegre, Susana Aranda-Verdú, Jose-Ignacio Zarzosa-López, Jorge Rubio-Climent, Eliseo Plasencia-Alcina, Antonio Pallarés-Sabater

**Affiliations:** 1 Department of Endodontics and Restorative Dentistry, School of Medicine and Dentistry, Catholic University of Valencia, Quevedo 2, 46001 Valencia, Spain; 2 Doctoral School, Catholic University of Valencia, 46001 Valencia, Spain

## Abstract

**Background:**

The aim of this paper was to evaluate the intratubular penetration percentage in the perimeter of the canals of the calcium silicate-based sealer HiFlow, using three warm obturation techniques, continuous wave (CW) and vertical condensation (VC) with two different types of gutta-percha (conventional (NG) and bioceramic-coated (BG), GuttaCore (GC) and single cone (SC) with BG in different root thirds.

**Material and Methods:**

180 human teeth with a single root were selected including incisors, canines and premolars were prepared and randomly divided into six groups (n=30). Teeth were filled using a bioceramic sealer TotalFill BC Sealer HiFlow (HiFlow) and two different types of gutta-percha, with CW, VC and GC techniques, the teeth in the control group were filled with SC technique and BG gutta-percha. The teeth were sectioned and evaluated as one-third portions in each case under a confocal laser microscope. The penetration ability in the canal’s perimeter was carried out with the Autocad® programme. Data was analyzed using Levene’s test (*p*<0,05), ANOVA test (*p*<0,05), Welch’s comparison test (*p*<0,05), Games-Howell multiple comparison test (*p*<0,05), Bonferroni test (*p*<0,05).

**Results:**

The percentages relative to penetration was higher in the warm obturation techniques than the SC in all thirds evaluated. Games-Howell test (*p*<0,05) showed up significant differences in multiple comparisons. There was greater penetration in the perimeter of the canals in the coronal third than in the apical third in all of the techniques.

**Conclusions:**

The warm obturation techniques (CW, VC and GC) generated a greater intratubular penetration percentage in the canal perimeter of the sealer than the single cone in all thirds.

** Key words:**HiFlow, calcium silicate-based sealer, confocal laser microscope, dentinal tubules.

## Introduction

The complete sealing and filling of the root canal system are essential because after chemomechanical preparation, the presence of microorganisms is detected. Sealers fill the irregularities of the root canal system and must therefore be applied. Furthermore, the sealing capacity is just as important as the antibacterial effect. The antibacterial effect of the bioceramic sealers may be achieved by direct contact action or a localized burial process; it is essential hence to distribute the sealer along the perimeter of the canal. Additionally, the use of a sealer creates a bond between the gutta-percha and the root dentine (1).

Bioceramic sealers boost the dentine remineralization processes, present acceptable cytotoxicity levels and offer a desirable degree of intratubular penetration (2). They are not prone to shrinkage, and therefore so the sealing capacity increases. In the work by Trope *et al*. (3), they detected evidence of expansion of bioceramic sealers in the setting reaction; they are characterized by having the ability to chemically bond to the dentine, so leakage decreases (4).

In the presence of biological fluids, calcium and phosphate ions present in the EndoSequence BC Sealer® (BC Sealer) may precipitate to form apatite (5). This ability is responsible for their bioactivity and excellent sealing capability (6). They also have antibacterial properties due to their high pH (7). The composition of the TotalFill BC Sealer HiFlow® (HiFlow) premixed calcium silicate–based sealers are made up of zirconium oxide, tricalcium silicate, dicalcium silicate, calcium hydroxide and fillers (8).

The intratubular penetration of the bioceramic sealer could generate a micromechanical interlock within the root dentine. In addition, the moisture that remains in the dentinal tubules could trigger their setting reaction with the production of hydroxyapatite, thus creating the aforementioned chemical bond with the root dentine (9). The micromechanical interlock and the chemical bond improve the resistance to any separation of the filling material and probably strengthens the root to prevent fractures (10).

Recently, the behaviour of the bioceramic sealers has been investigated when they have been exposed to heat application. The chemophysical properties were investigated during or shortly after heat exposure (11). While the physical properties of the new bioceramic sealer HiFlow was not adversely affected by heat, a negative modification of the properties in the older bioceramic sealers was observed (12).

The bioceramic-coated gutta-percha points (BG) is a modification of the inner composition of the gutta-percha cone and the coating of the outer surface with calcium silicate nanoparticles. According to the manufacturer, these types of points of gutta-percha should be used together with a bioceramic sealer.

The aim of this paper was to evaluate the intratubular penetration percentage in the perimeter of the canals of the calcium silicate-based sealer HiFlow, using three warm obturation techniques, continuous wave (CW) and vertical condensation (VC) with two different types of gutta-percha (conventional (NG) and BG), GuttaCore (GC) and single cone (SC) with BG in different root thirds. The null hypothesis states there are no differences between the intratubular penetration percentage in the perimeter of the canals obtained for each of the obturation techniques.

## Material and Methods

This piece of research was approved by the Research Ethics Committee of UCV, (Registration number: UCV/2019-2020/001.).

-Selection of samples

To carry out the study, 180 human teeth with a single root were selected (including incisors, canines and premolars). The teeth were extracted for periodontal reasons. Roots with acute curvatures, immature apex, resorption, fissures, calcification, previous endodontic treatment or initial apical sizes larger than 15 were rejected. After extraction, the teeth were immersed for one hour in a 5.25% sodium hypochlorite solution after which the root surfaces were cleaned with a Gracey® 1-2 curette (Hu-Friedy, USA) and then stored in a saline solution.

Root canal preparation

Two preoperative X-rays were taken in two views to check the presence of a single canal. Buccolingual and mesiodistal parallel radiographs were obtained for each tooth. After opening the root canal system with a tapered cone burr (Komet, Lemgo, Germany) and constant irrigation, the canal was located with a DG16® endodontic probe (Hu-Friedy, USA). The root of the clinical crown was separated at the amelocemental junction with a handpiece diamond disc and water cooling; a size 10 or 15 K file was then introduced into the canal space, the working length (WL) was established 0.5 mm from the apical foramen by visual observation.

All canals were prepared with Protaper Gold® (Dentsply Maillefer, Ballaigues, Switzerland) according to the producer’s instructions. The shaping files S1 (250 rpm and 3 Ncm) and S2 (250 rpm and 1 Ncm) were used with circumferential movements and brushing at the working length, while the finishing files F1 (250 rpm and 1.5 Ncm) and F2 (250 rpm and 2.5 Ncm), were used with a pecking motion with the Gold ReciprocTM motor (VDW, Munich, Germany). After each file was used, the canal was flushed out with 5.25% NaOCl solution. The permeability of the canals was checked by inserting a size 10 file through the apical foramen after instrumentation was complete.

As the final irrigation protocol, canals were irrigated for 1 minute with 5 ml of 5.25% sodium hypochlorite, 1 minute with 5 ml of 17% EDTA, and 30 seconds with 5 ml of chitosan-hydroxyapatite precursor, 10 ml of saline solution was used for a final flush out and also used in the established order of different irrigants (13,14). The irrigants were activated using the EDDY® sonic tip system (VDW, München, Germany) with Air Scaler. The canals were dried with F2 paper tips. This chemomechanical sample preparation procedure was common denominator, regardless of the obturation technique used.

-Obturation of the root canals

0,1% of Rhodamine BTM (Sigma-Aldrich Corp., USA) was added to the bioceramic sealer in relation to the weight for its subsequent observation through the confocal laser microscope, thanks to the fluorescent property of the dye.

The samples were then randomly divided into 6 experimental groups (n=30). The samples were sealed with the different obturation techniques set forth as follows:

•Group 1: SC with TotalFill BC Points® BG and HiFlow.

•Group 2: GC technique with HiFlow.

•Group 3: CW technique with Protaper F2® gutta-percha, NG pellets and HiFlow.

The teeth were filled using the CW technique, designed by Buchanan (15). The plugger was checked with the rubber stopper positioned at less than 4 mm from the working length. The shutter unit used was E&Q Master® (Meta Biomed, Chalfont, PA, USA), at a temperature of 220ºC for the hot plugger and a temperature of 200ºC corresponding to the warm gutta-percha injection unit.

•Group 4: CW technique with TotalFill BC Points® BG, BG pellets and HiFlow.

•Group 5: VC technique with Protaper F2® gutta-percha, NG pellets and HiFlow.

The teeth were filled using the VC technique, designed by Schilder. We use the System-B® obturation unit (Sybron Dental, Orange, CA, USA) at a temperature of 100°C in the hot plugger, removing 2-3mm portions of gutta-percha and condensing it until reaching 4mm of the working length, and a temperature of 200ºC for the warm gutta-percha injection unit.

•Group 6: VC technique with TotalFill BC Points® BG, BG pellets and HiFlow®.

-Specimen preparation

Once all the samples were sealed, they were stored at 37ºC and 100% humidity in a laboratory incubator for 14 days to allow complete sealer setting. The root was divided into three parts, taking a sample from each third: the coronal, middle and apical third (the apical third was taken by subtracting a length of two millimetres from the root apex). Horizontal cuts were made using a 0,3 mm diamond disc handpiece with water cooling (16), 1 mm thick slices were then obtained; the slices were polished with Soft Lex discs (3M (™) ESPE (™) St. Paul, MN, USA). After observation with the confocal laser microscope (Leica TCS SP8 Confocal Microscope) and the 5x object lens, photographs of each of the samples were taken for analysis and studied.

**Figure 1:**
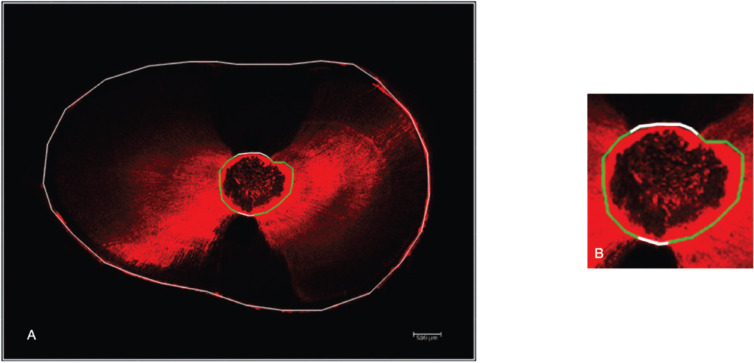
A. The external White line outlines the tooth. B. It denotes the perimeter of the canal; in the White lines there is no evidence of sealer penetration. In the Green lines there is sealer penetration.

The intratubular penetration percentage in the perimeter of the canals of the sealer were carried out with AutoCad® Software from the images obtained and collected in a data sheet. Firstly, each image was scaled to 500 µm in order to obtain a correct measurement of all its elements. The appropriate AutoCad tool function was applied to the perimeter of the canals to obtain the intratubular penetration percentage (Fig. 1). The perimeter of the canal with tubular penetration was divided by the total canal perimeter and multiplied by 100 (17).

All measurements were recorded by one of the authors. In case of doubt on first viewing, the sample was polished and a new image was obtained. All data was recorded, and then analyzed.

-Statistical analysis

The statistical analysis of the data collected for the present study was carried out using SPSS 23 software using a confidence level of 95% and considering them statistically significant (*p*<0,05). As the sample size is sufficiently large, (n=30), we used parametric methods of comparison. Levene’s test, ANOVA test (middle and apical third), Welch’s comparison test (coronal third), Games-Howell multiple comparison test (coronal third), Bonferroni test (middle and apical third) were used to evaluate the percentage of sealer penetration in the canal perimeter.

## Results

**Figure 2:**
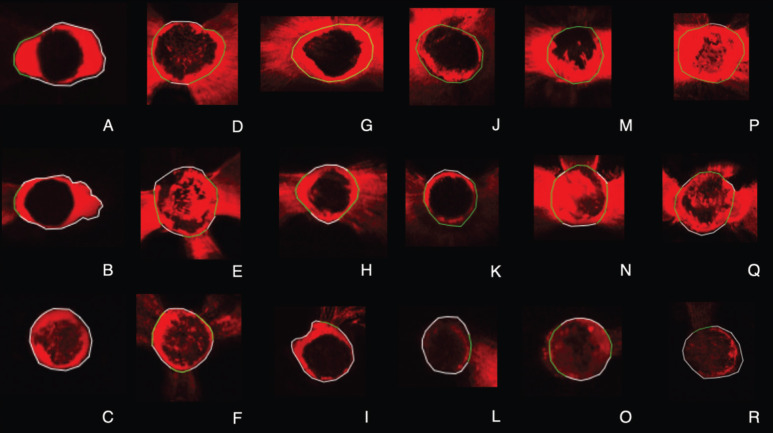
Confocal images of representattive setions. Single Cone: A (cronal), B (middle), C (apical). GuttaCore: D (coronal), E (middle), F (apical). Continuonus wavw with conventional gutta-percha: G (coronal), H (middle), I (apical). Continuonus wave with bioceramic-coated gutta-percha: J (coronal), K (middle), L (apical). Vertical condensation with conventional gutta-percha: M (coronal), N (middle), O (apical). Vertical condensation with bioceramic-coated gutta-percha: P (coronal, Q (middle), R (apical).

The study showed the average of intratubular penetration percentage in the perimeter of the canals of the sealer (Table 1). Figure 2 demonstrated the representative samples of confocal images of the different groups and thirds.

**Table 1:**
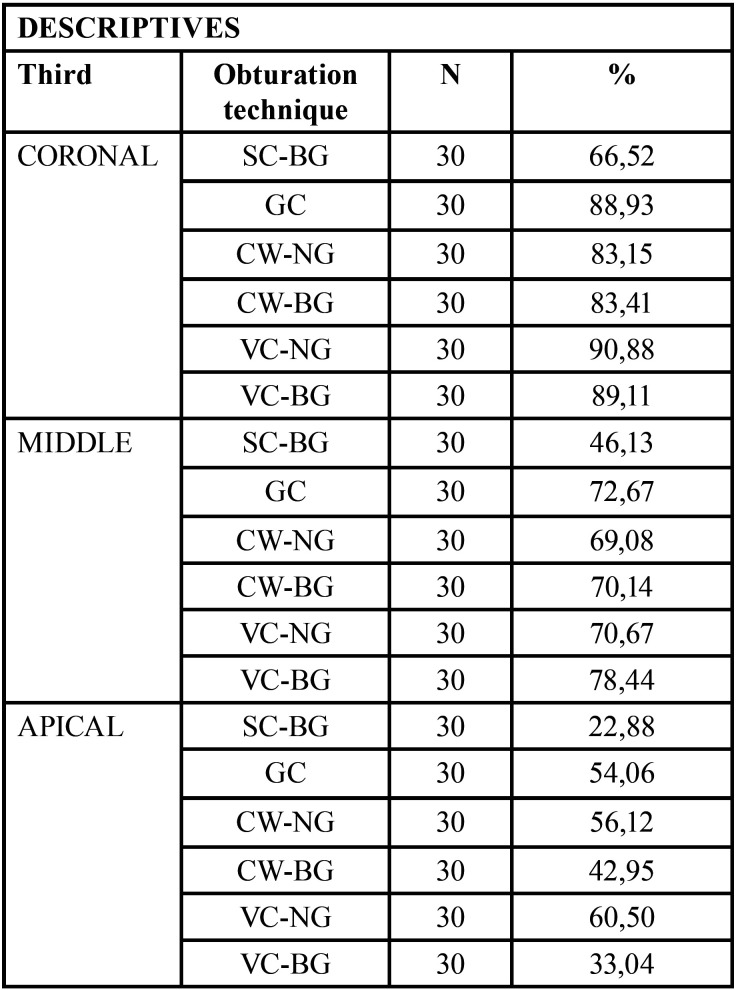
Average of intratubular penetration percentage in the perimeter of the canals (%).

The results of Levene’s test (Table 2) showed that the middle (*p*= 0,106) and apical (*p*= 0,141) third was greater than 0,05. For this reason, we used the ANOVA test (Table 3) in order to study the differences between the intratubular penetration percentage of each technique. In the coronal third (*p*= 0,014) we used Welch’s comparison test (Table 3).

**Table 2:**
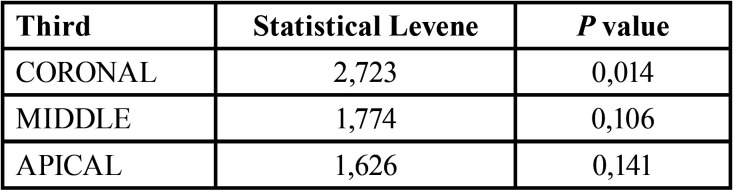
Levene’s test.

**Table 3:**
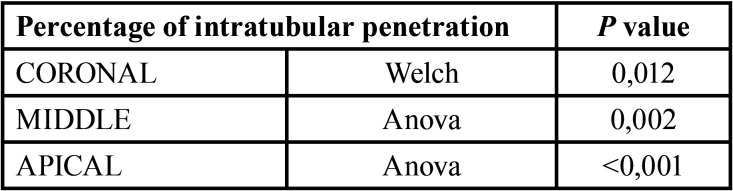
Welch’s comparison test and ANOVA Test.

In all thirds (*p*<0,05), there was a statistical difference between at least two of the obturation techniques. In order to study these differences, we used the Games-Howell multiple comparison test (Table 4) in the coronal third. In the middle and apical third, we used the Bonferroni test (Table 4).

**Table 4:**
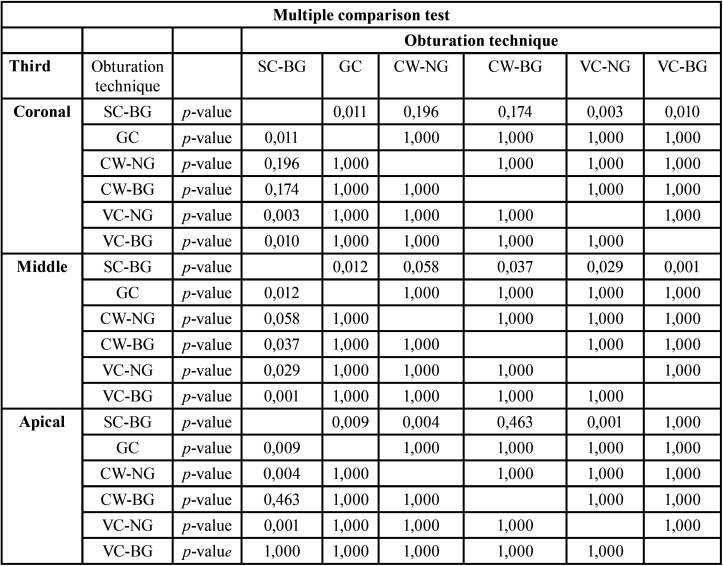
Games-Howell Test and Bonferroni Test.

In Table 4, the bold type numbers indicate the statistical differences between the different obturation techniques.

## Discussion

In the present study, we used the confocal laser microscope, since the preparation of specimens destined for the scanning electron microscope may lead to a loss of sealer and deformation of the sample (18).

Rhodamine B could be suiTable with the bioceramic sealers, because the narrow amount (0.1%) used did not modify the sealer’s qualities (19). The sample cuts were performed in the horizontal plane as the dentine of the root canal cannot be completely observed in the longitudinal plane (17).

The intratubular penetration percentage in the perimeter of the canals suggested a highly clinical significance level (20). This penetration performance provides a physical barrier to the entry or exit of micro-organisms into the canal regardless of the depth of penetration or the area penetrated. Furthermore, a bactericidal effect is created by contact action between the sealer and the bacteria through its antibacterial effect (21). These two properties (contact action and physical barrier) are favourable for the healing of the periapical lesion. The major contact surface of the sealer with the canal walls determines the sealing of the root dentine (18). Few studies have assessed this parameter.

The results of our study showed a large percentage of penetration sealer in the warm obturation techniques compared to the SC technique. In general, the heat resulted in a positive effect in terms of penetration percentage. A statistical difference showed up in the different techniques although not in all cases. These varying results may be due to the different factors that affected the penetration ability of the sealer (root third, properties of the sealer, obturation technique, irrigation, instrumentation…).

Wang *et al*. (22) evaluated percentage sealer penetration with two different sealers (iRoot and AH Plus) and using the SC and VC techniques at 2-4 and 6 mm. There were no statistical differences between groups SC and AH Plus and VC with AH Plus and between the SC and VC groups with iRoot. At 2 mm, more penetrated segments of the root canal were observed in the iRoot groups than in the AH Plus groups. At the horizontal levels of 4 and 6 mm respectively, there were no statistically significant differences in the penetrated segment of root canal between these four groups. The differences with our outcomes may be due to the use of the specific techniques not being appropriate for these sealers. The study of Chen *et al*. (11) concluded that Endosequence BC sealer is not adequate for warm obturation techniques. In the study by Trope *et al*. (3) the resin sealer was observed to undergo shrinkage with the setting, so they were not suiTable for the SC technique. The major apical diameter may have been influenced in the best access to the irrigants in the apical third and may have modified the results.

McMichael *et al*. (19) compared SC and CW technique with different sealers (BC Sealer, MTA Fillapex, NeoMTA Plus and QuickSet2). At 1 mm, the outcomes were lower in penetration percentage than at 5 mm. At the 5-mm level, there was no significant difference in percentage of sealer penetration between the VC or SC technique between any of the sealers. The difference with our results in the percentage of penetration may be due to the different instrumentation, the non-activation of the irrigants and the lower sample. In addition, the cuts were standardized at 5 mm in the study by McMichael; in our study however, the cuts were made by dividing the root into thirds. More outliers were measured for the SC technique than for the rest of the techniques in both studies.

Sealer penetration percentage was significantly higher, at the 5 mm distance (middle third) compared with the 1 mm (apical third) with the warm obturation technique. These outcomes fall in line with the findings of our study; an explanation may be that tubular density and diameter tend to decline in apical thirds. In addition, it is difficult to transport the irrigants to the apical third in order to remove the smear layer of the dentinal tubules.

One of the interactions observed in our study was the so-called mineral infiltration zone (MIZ) which is a hybrid zone where hydroxyapatite recrystallisation occurs in dentine when a calcium silicate-based sealer is applied (23). These reactions were unexpectedly discovered when dentine tubules were converted into homogeneous structures due to by hydroxyapatite recrystallisation. However, such MIZ behaviour was not observed in all samples. MIZ has not been shown to positively or negatively affect the outcome of endodontic treatment (18). However, further studies would be required to determine the influence of MIZ on root canal treatment.

In some of the samples scanned by confocal laser microscopy, the penetration of the sealer into the dentinal tubules was not homogeneous. The varying directions of the dentinal tubules may affect the results (24). Sealer penetration was found to be higher in the buccolingual direction than in the mesiodistal direction, although not in all of the samples analyzed. This may be due to increased sclerosis in the dentinal tubules located on the mesial and distal sides of the canal lumen, with greater buccolingual than mesiodistal penetration observed. It is common in the single-root teeth over a broad age-range (25). Areas of sclerotic dentine are more common in the apical third (26).

The results of this study showed that the intratubular penetration percentages in the canal perimeter of the sealer, independently of the technique used, were greater in the coronal section compared to the apical section. One explanation for this finding could be due to a higher efficiency of irrigant administration and smear layer removal at the coronal levels. The smear layer sticks to the canal walls, forms physical barriers and creates contamination in the dentinal tubules, blocking sealer penetration (27). In addition, tubular diameter, density and number decrease at the apical levels, which explains the tendency for sealer penetration to decrease from the coronal to the apical region (20). In addition, the viscosity and flow of endodontic sealers may determine the efficiency with which they penetrate the dentinal tubules. Chen *et al*. (11) showed that HiFlow had a higher flow than BC Sealer at higher temperatures.

It is important to create an adequate glide path to disinfect properly before obturating the apical third. Due to the morphological characteristics of the tooth, it is difficult to deliver irrigant and sealer. We must consider whether the taper of the master apical file allows these minimum criteria for disinfection and obturation to be adequately met. Apical preparation using 2 sizes larger than the initial apical binding file with a taper of 4% is insufficient and results in significantly lower success rates compared to larger preparation sizes and taper (28).

The sealers penetrated into the dentinal tubules can maintain their bactericidal effect (29) and therefore favourable for the healing of the periapical lesion.

In conclusion, within the limitations of this study, for each type of gutta-percha and technique, dentinal tubule penetration was higher in the coronal section than in the apical section. The warm obturation techniques (continuous wave, vertical condensation and Guttacore) showed more intratubular penetration percentage in the canal perimeter of the sealer than in the single cone in all of the thirds studied.

## References

[1] Nielsen BA, Beeler WJ, Vy C, Baumgartner JC (2006). Setting times of Resilon and other sealers in aerobic and anaerobic environments. J Endod.

[2] Vouzara T, Dimosiari G, Koulaouzidou EA, Economides N (2018). Cytotoxicity of a New Calcium Silicate Endodontic Sealer. J Endod.

[3] Trope M, Bunes A, Debelian G (2015). Root filling materials and techniques: bioceramics a new hope?. Endodontic Topics.

[4] Jardine AP, Rosa RA, Santini MF, Wagner M, So MV, Kuga MC (2016). The effect of final irrigation on the penetrability of an epoxy resin-based sealer into dentinal tubules: a confocal microscopy study. Clin Oral Investig.

[5] Yadav S, Nawal RR, Chaudhry S, Talwar S (2020). Assessment of Quality of Root Canal Filling with C Point, Guttacore and Lateral Compaction Technique: A Confocal Laser Scanning Microscopy Study. Eur Endod J.

[6] Zamparini F, Siboni F, Prati C, Taddei P, Gandolfi MG (2019). Properties of calcium silicate-monobasic calcium phosphate materials for endodontics containing tantalum pentoxide and zirconium oxide. Clin Oral Investig.

[7] Lovato KF, Sedgley CM (2011). Antibacterial activity of endosequence root repair material and proroot MTA against clinical isolates of Enterococcus faecalis. J Endod.

[8] Antunes TBM, Janini ACP, Pelepenko LE, Abuna GF, Paiva EM, Sinhoreti MAC (2021). Heating stability, physical and chemical analysis of calcium silicate-based endodontic sealers. Int Endod J.

[9] Han L, Okiji T (2013). Bioactivity evaluation of three calcium silicate-based endodontic materials. Int Endod J.

[10] Ghoneim AG, Lutfy RA, Sabet NE, Fayyad DM (2011). Resistance to fracture of roots obturated with novel canal-filling systems. J Endod.

[11] Chen B, Haapasalo M, Mobuchon C, Li X, Ma J, Shen Y (2020). Cytotoxicity and the Effect of Temperature on Physical Properties and Chemical Composition of a New Calcium Silicate-based Root Canal Sealer. J Endod.

[12] Aksel H, Makowka S, Bosaid F, Guardian MG, Sarkar D, Azim AA (2021). Effect of heat application on the physical properties and chemical structure of calcium silicate-based sealers. Clin Oral Investig.

[13] Zeng C, Willison J, Meghil MM, Bergeron BE, Cutler CW, Tay FR (2018). Antibacterial efficacy of an endodontic sonic-powered irrigation system: An in vitro study. J Dent.

[14] Neelakantan P, Ounsi HF, Devaraj S, Cheung GSP, Grandini S (2019). Effectiveness of irrigation strategies on the removal of the smear layer from root canal dentin. Odontology.

[15] Buchanan LS (1998). Continuous wave of condensation technique. Endod Prac.

[16] Faus-Llácer V, Collado-Castellanos N, Alegre-Domingo T, Dolz-Solsona M, Faus-Matoses V (2015). Measurement of the percentage of root filling in oval-shaped canals obturated with Thermafil Obturators and Beefill 2in1: In vitro study. J Clin Exp Dent.

[17] Eymirli A, Sungur DD, Uyanik O, Purali N, Nagas E, Cehreli ZC (2019). Dentinal Tubule Penetration and Retreatability of a Calcium Silicate-based Sealer Tested in Bulk or with Different Main Core Material. J Endod.

[18] Jeong JW, DeGraft-Johnson A, Dorn SO, Di Fiore PM (2017). Dentinal Tubule Penetration of a Calcium Silicate-based Root Canal Sealer with Different Obturation Methods. J Endod.

[19] McMichael GE, Primus CM, Opperman LA (2016). Dentinal Tubule Penetration of Tricalcium Silicate Sealers. J Endod.

[20] Bolles JA, He J, Svoboda KK, Schneiderman E, Glickman GN (2013). Comparison of Vibringe, EndoActivator, and needle irrigation on sealer penetration in extracted human teeth. J Endod.

[21] Akcay M, Arslan H, Durmus N, Mese M, Capar ID (2016). Dentinal tubule penetration of AH Plus, iRoot SP, MTA fillapex, and guttaflow bioseal root canal sealers after different final irrigation procedures: A confocal microscopic study. Lasers Surg Med.

[22] Wang Y, Liu S, Dong Y (2018). In vitro study of dentinal tubule penetration and filling quality of bioceramic sealer. PLoS One.

[23] Atmeh AR, Chong EZ, Richard G, Festy F, Watson TF (2012). Dentin-cement interfacial interaction: calcium silicates and polyalkenoates. J Dent Res.

[24] Aydin ZU, Ozyurek T, Keskin B, Baran T (2019). Effect of chitosan nanoparticle, QMix, and EDTA on TotalFill BC sealers' dentinal tubule penetration: a confocal laser scanning microscopy study. Odontology.

[25] Russell AA, Chandler NP, Hauman C, Siddiqui AY, Tompkins GR (2013). The butterfly effect: an investigation of sectioned roots. J Endod.

[26] Ribeiro RG, Marchesan MA, Silva RG, Sousa-Neto MD, Pécora JD (2010). Dentin permeability of the apical third in different groups of teeth. Braz Dent J.

[27] Kuci A, Alacam T, Yavas O, Ergul-Ulger Z, Kayaoglu G (2014). Sealer penetration into dentinal tubules in the presence or absence of smear layer: a confocal laser scanning microscopic study. J Endod.

[28] Fatima S, Kumar A, Andrabi S, Mishra SK, Tewari RK (2021). Effect of Apical Third Enlargement to Different Preparation Sizes and Tapers on Postoperative Pain and Outcome of Primary Endodontic Treatment: A Prospective Randomized Clinical Trial. J Endod.

[29] Branstetter J, von Fraunhofer JA (1982). The physical properties and sealing action of endodontic sealer cements: a review of the literature. J Endod.

